# Occurrence of the South American Tomato Leaf Miner, *Tuta absoluta* (Meyrick) in Southern Shan, Myanmar

**DOI:** 10.3390/insects12110962

**Published:** 2021-10-22

**Authors:** Sopana Yule, Ni Ni Htain, Aung Kyaw Oo, Paola Sotelo-Cardona, Ramasamy Srinivasan

**Affiliations:** 1 World Vegetable Center, Kamphaeng Saen Campus, East and Southeast Asia Research and Training Station Kasetsart University, Nakhon Pathom 73140, Thailand; sopana.yule@worldveg.org; 2 Plant Protection Division, Department of Agriculture, Ministry of Agriculture, Livestock and Irrigation, Yangon 11011, Myanmar; ninihtain@gmail.com (N.N.H.); aungkyaw.flower@gmail.com (A.K.O.); 3 World Vegetable Center, Shanhua, Tainan 74151, Taiwan; paola.sotelo@worldveg.org

**Keywords:** *Tuta absoluta*, Inle Lake, land cultivation, floating tomato cultivation, Southeast Asia

## Abstract

**Simple Summary:**

Tomato is the most important vegetable grown in Myanmar. However, its production is threatened by the invasion of the new insect pest, the South American tomato leaf miner, *Tuta absoluta.* Preliminary surveys on pest occurrence on tomato crops in Myanmar suggested the presence of *T. absoluta* in Southern Shan State, but there was no official survey conducted until the end of 2019. Therefore, this study aimed to confirm the presence of *T. absoluta* in Myanmar. *Tuta absoluta* presence was confirmed in all fields and locations surveyed in Myanmar, under two cultivation methods, (i.e., floating-and land cultivation). Higher infestation levels recorded at theKalaw location (land cultivation) seemed to correspond with plants at flowering and early harvesting stages. Moreover, information collected from the survey showed that the amount of *T. absoluta* larvae was significantly higher in the lower third of the plants followed by the middle section and upper section, respectively, in three fields of the survey.

**Abstract:**

The South American tomato leaf miner, *Tuta absoluta* (Meyrick) (Lepidoptera: Gelechiidae), one of the most important invasive insect pests affecting tomato production worldwide, was for the first time detected in Myanmar. Preliminary surveys on pest occurrence on tomato crops in Myanmar suggested the presence of *T. absoluta* in Southern Shan State, but there was no official survey conducted until the end of 2019. Therefore, this study aimed to confirm the presence of *T. absoluta* in Myanmar. The presence of *T. absoluta* was specifically observed in the Southern Shan State, which is the largest tomato production area in Myanmar, where tomato is grown under two cultivation methods, floating and land cultivation. The highest *T. absoluta* infestation was recordedat Kalaw with (82%), followed by Inle Lake (i.e., floating cultivation) (20%) and Pin Ta Ya (10%). The amount of *T. absoluta* larvae was significantly higher in the lower third of the plants, followed by the middle section and upper section, respectively, in three fields surveyed. Potential and severe economic damage may be expected if management practices are not in place to reduce the presence of this invasive pest. It is of immediate importance that plant protection and quarantine offices of ASEAN member states coordinate their response to *T. absoluta* and build their capacity to monitor the pest and develop a strategy for when it arrives. In addition, a suitable management strategy is needed to reduce the occurrence of this invasive pest.

## 1. Introduction

Tomato is the most important vegetable grown in Myanmar, with three times more area harvested compared to the second and third vegetable crops, mustard and cabbage, respectively [1]. The production in Myanmar occupied over 112,000 ha and 15 t/ha productivity in 2015–2016, with the major cultivation areas located in Sagaing Division, Magway Division, Mandalay Division and Shan State. Southern Shan State is one of the important tomato production areas in Myanmar, with over 6000 ha [2]. In Shan State, tomatoes are grown under two different cultivation methods, viz., floating- and land cultivation, with a large wholesale market of tomato located at Inle Lake. Despite the large harvested area, tomato yields are one-third of the world average [1]. In general, the presence of insect pests and diseases constrain tomato production worldwide. The South American tomato leaf miner, *Tuta absoluta* (Meyrick) (Lepidoptera: Gelechiidae) is one of the most important invasive insect pests affecting tomato production worldwide in the recent decade [3]. *T. absoluta* is native to Peru in South America and since early 2000s, it has spread through the rest of America, Africa, Europe and Asia. In 2014, the presence of *T. absoluta* was officially registered in two states of India [4]. Subsequently, Bangladesh, Nepal, and several countries in Central Asia had also reported the presence of this pest. In contrast, detections of this insect have not been announced in North America and some parts of Asia such as Cambodia, Japan, Laos, Indonesia and Thailand [5,6,7,8,9]. To the best of our knowledge, there are no official reports of *T. absoluta* presence in most Southeast Asian countries [9,10]. However, since *T. absoluta* has been already detected throughout India and Central Asia, there is a high possibility that sooner or later this pest will spread across borders to tomato production areas in Southeast Asian countries. *Tuta absoluta* larva feeds on the mesophyll of aerial parts of the plant and creates mine blotches on leaves, stems, buds and fruits [11,12]. This insect feeds primarily on tomato (*Solanum lycopersicum* L.), but other solanaceous and non-solanaceous crops including potato (*Solanum tuberosum* L.), eggplant (*Solanum melongena* L.), melon pear (*Solanum muricatum* Aiton), spinach (*Spinacia oleracea* L.), alfalfa (*Medicago sativa* L.), beet (*Beta vulgaris* L.), watermelon (*Citrullus lanatus* (Thunberg) Matsumura & Nakai) and common bean (*Phaseolus vulgaris* L.) can also serve as secondary hosts [13,14,15]. If management strategies are not in place, direct economic losses can be up to 80–100% on tomato production [14,16].

Preliminary surveys on pest occurrence on tomato production in Pin Ta Ya and Nyaungshwe, Myanmar, conducted by the Plant Protection Division (PPD) in 2017–2018 suggested the presence of *T. absoluta* in Southern Shan State, but there was no official survey conducted until the end of 2019. Therefore, this study aimed to confirm the presence of *T. absoluta* in Myanmar, and then to evaluate the influence of floating- and land cultivation of tomatoes on *T. absoluta* infestation levels in Southern Shan State. Information provided in this study will help in confirming the presence of *T. absoluta* in the region and assist in the development of plant protection and quarantine strategies to coordinate the response to reduce the spread and infestation of *T. absoluta*.

## 2. Materials and Methods

### 2.1. Survey Location

Three locations in different cultivation regions in Southern Shan State, Myanmar, were surveyed for the presence of *T. absoluta* in December 2019. One of the locations, Nyaungshwe, is known for having a floating cultivation method, whereas, the other two locations—Kalaw and Pin Ta Ya—have a conventional land cultivation method (Table 1). Within each location, three fields were selected and surveyed for *T. absoluta* infestation percentage and population density. To determine the infestation percentage, 20 plants in each field were randomly selected; plants were in different developmental stages, comprising flowering stage (35–40 days), early harvesting stage (3.5 months) and last harvesting stage (4–5 months). Number of damaged leaflets and total number of leaflets were recorded. In addition, the number of larvae and mine blotches per leaf were counted on three leaflets from upper, middle and lower strata of the plant. The leaf infestation percentage was calculated as:

Leaf infestation (%) = [(No. of infested leaves/total No. of leaves) × 100]

### 2.2. Statistical Analysis

Statistical analysis was performed for the number of larvae and mine blotches data in order to determine *T. absoluta* oviposition preferences on upper, middle and lower leaflets within a plant for each field and location surveyed. To adjust data as normal distribution, data was square-root transformed (*sqrt x*+0.5), and later analyzed using Proc GLM of SAS, version 9.4 (SAS Institute, Cary, NC, USA). Non-transformed means are used in the tables. Furthermore, correlations between *T. absoluta* larvae and mine blotches were calculated using the procedure Proc CORR of SAS version 9.4 (SAS Institute, Cary, NC, USA) for different plant positions (upper, middle and lower section of the plants) and across locations and fields in order to have general information for the prediction of *T. absoluta* presence based on mine blotches or vice versa.

## 3. Results

### 3.1. Tuta absoluta Survey

**Figure 1:**
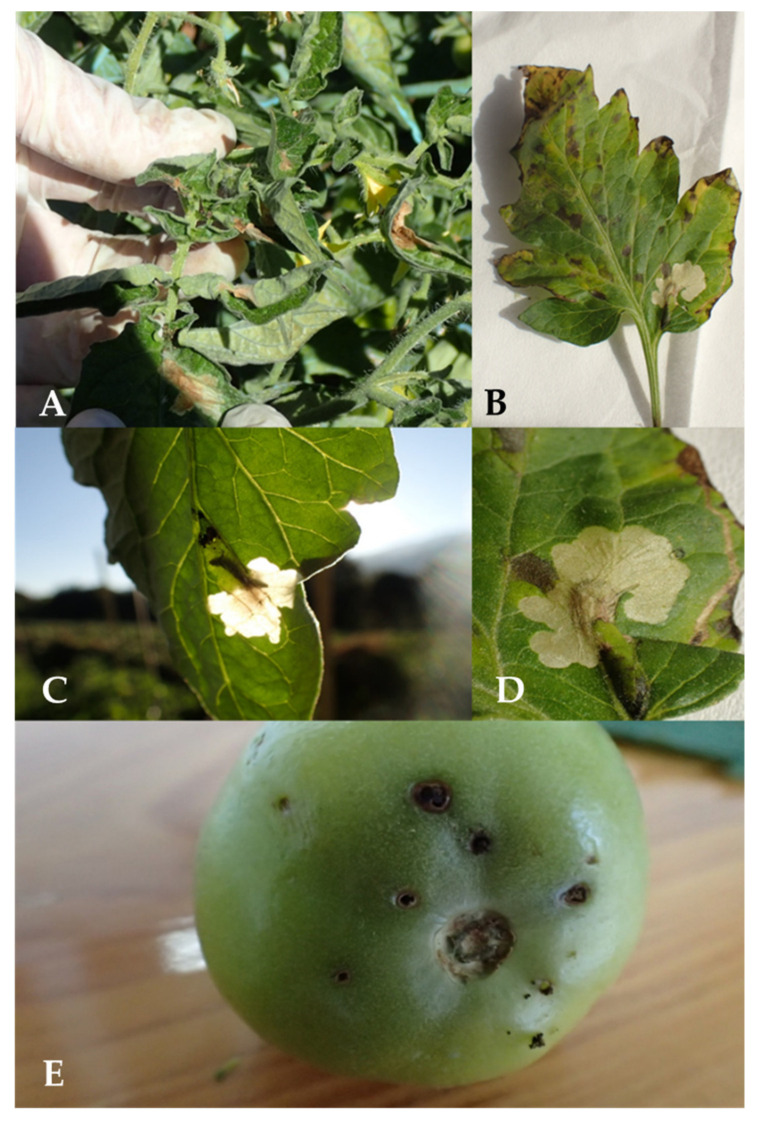
Typical damage on tomato leaves (**A**–**D**) and fruits (**E**) caused by the larvae of *Tuta absoluta*.

**Figure 2:**
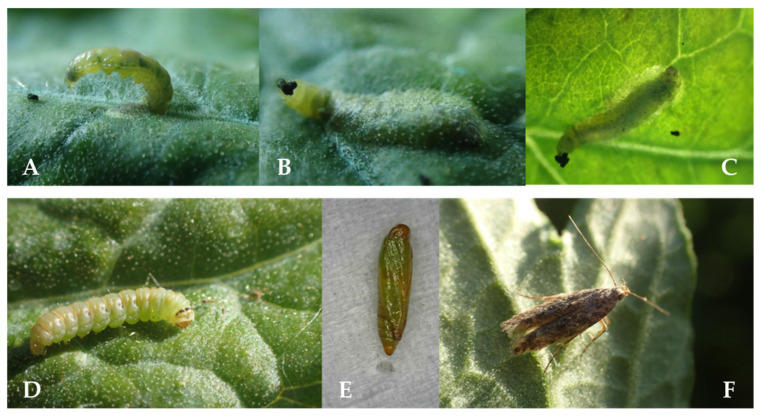
*Tuta absoluta* development stages: larva (**A**–**D**), pupa (**E**) and adult (**F**). Details of larval mining under leaf surface on **A**–**C**.

*Tuta absoluta* was recorded in Southern Shan State in December 2019 (Figure 1 and Figure 2). This state is the largest tomato production area in Myanmar, where tomato is grown under two different cultivation methods—floating- and land cultivation. The floating cultivation is typically found at Inle Lake, Nyaugshwe, where farmers grow tomato year-round. Tomato plants in the selected fields were found in different stages including flowering, early harvesting and late harvesting stages. The highest infestation was recorded for Kalaw (i.e., land cultivation 1) with 82% infestation, followed by Inle Lake (i.e., floating cultivation), with 20% infestation, and Pin Ta Ya (i.e., land cultivation 2), with 10% infestation (Table 2).The two highest infestation levels were recorded at the Kalaw location for early harvesting plants (approx. 3.5-month-old plants), followed by flowering stage plants (approx. 35–45-day-old plants) in fields 2 and 3, respectively (Table 2).The lowest infestation levels (5%) were observed at the Pin Ta Ya location (all three fields) for plants in early flowering (approx. 35-day-old plants) and late harvesting (4.5–5-month-old plants) stages (Table 2). In addition, late harvesting plants (4-month-old plants) in field 3 of Inle Lake also sustained low level infestations.

In line with the infestation levels, the highest number of larvae and mines were also observed in fields 2 and 3 at the Kalaw location (Table 2). Infestation levels and number of mine blotches were also consistent across regions, with Kalaw presenting four- and seven-times higher number of mines compared to Inle Lake and Pin Ta Ya, respectively (Table 2).

### 3.2. Tuta absoluta Leaflet Preferences

**Figure 3:**
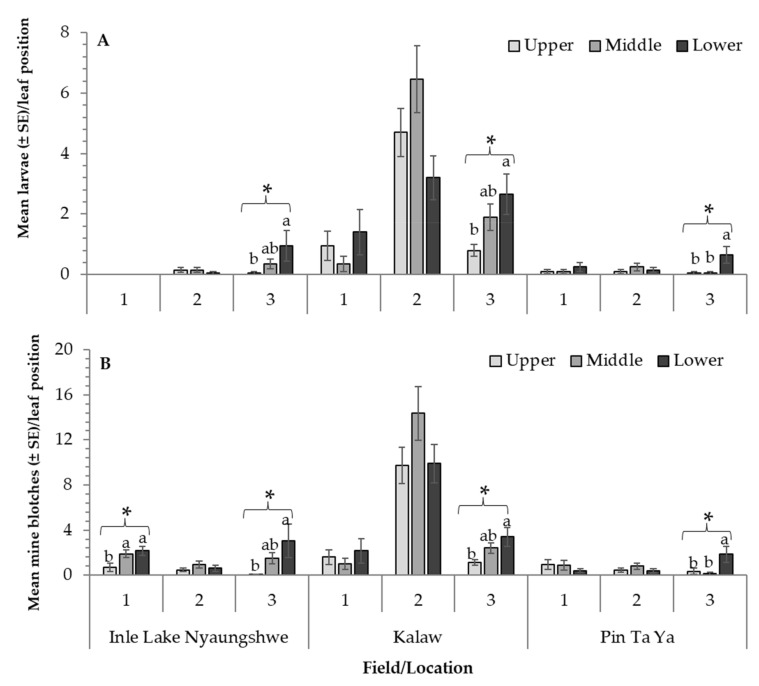
(**A**) Mean *Tuta absoluta* larvae and (**B**) mine blotches found on upper, middle and lower strata recorded in three locations and two tomato cultivation methods in Myanmar. * Statistical values provided for the locations where *Tuta absoluta* larvae and mine blotches differed among leaf position. Bars with the same letter(s) within each field/location are not significantly different (*p* < 0.05) (See text for statistical details).

Information collected during the survey was used to recognize potential preferences of *T. absoluta* in terms of leaf positions (i.e., upper third, middle third or lower third of the plant). The number of *T. absoluta* larvae was significantly higher in the lower third of the plant followed by the middle section and upper section, respectively, in three fields of the survey (i.e., field 3 Inle Lake (F_2, 59_ = 3.19; *p* =0.0487), field 3 Kalaw (F_2, 59_ = 4.25; *p* =0.0191) and field 3 Pin Ta Ya (F_2, 59_ = 5.09; *p* =0.0093)) (Figure 3A). Similarly, the amount of mine blotches found in the lower section of the plant was higher compared to those recorded in the middle and upper section, respectively, in four fields of the survey (i.e., fields 1 (F_2, 59_ = 5.79; *p* =0.0051) and 3 (F_2, 59_ = 4.61; *p* =0.0139) of Inle Lake, field 3 Kalaw (F_2, 59_ = 4.96; *p* =0.0103) and field 3 Pin Ta Ya (F_2, 59_ = 4.61; *p* =0.0139)) (Figure 3B).

The correlations between *T. absoluta* larvae and mine blotches across field and locations were highly significant for all three plant strata. The correlation values between the two factors in the upper leaves was r = 0.8996; *p* <0.0001; *n* =180; middle section: r = 0.8389; *p* <0.0001; *n* =180; and lower section: r = 0.7896; *p* <0.0001; *n* =180.

## 4. Discussion

This survey confirmed the presence of *Tuta absoluta* in all fields and locations surveyed in Myanmar, and under two cultivation methods, (i.e., floating- and land cultivation). Furthermore, higher infestation levels recorded at the Kalaw location seemed to correspond with plants at flowering and early harvesting stages. High infestation levels indicate a potential of severe economic damage if management practices are not initiated in a timely manner to reduce the occurrence of this invasive insect pest. According to Diatte et al. (2018) [17], *T. absoluta* starts to colonize on tomato just after transplanting at vegetative stage and reaches a maximum infestation during the flowering–fruiting stages, with 87.50% infestation detected in early fruiting stage, followed by early flowering-, vegetative- and harvesting stages. In line with this, Allache et al. (2015) [18] indicated that the number of *T. absoluta* eggs and larvae were very low in early vegetative stage but increased in late harvest stages. Our observation was also in agreement with Abdelhady et al. (2020) [19], who found that the attraction of *T. absoluta* to tomato plants varied with the age of the plants, with high percentages of attraction associated with the 30- and 45-day-old plants. Additionally, we also observed that most tomato plants in Nyaungshwe and Pin Ta Ya at late harvesting stage were severely infected by diseases such as late blight (*Phytophthora infestans*) and leaf spot (*Corynespora cassiicola*) [20]. We believe that given the disease prevalence, *T. absoluta* moths might have preferred to choose healthy plants or may also have preferred young plants for oviposition, but this hypothesis requires further validation.

The number of *T. absoluta* larvae was significantly higher in the lower stratum of the plant, followed by the middle stratum and upper stratum, respectively, in three fields of the survey. Previous studies have found that *T. absoluta* females laid their eggs on the upper and middle strata of the plants, and as the plants grow, the larvae is then found in the lower and middle strata of the plant, as the infested tissues were part of the upper and middle parts of the plant [21,22]. With the information gathered during the survey, we also found high correlation between the number of mine blotches and larvae across fields, locations and for all plant sections evaluated. This information is important in terms of monitoring the presence of *T. absoluta* in the fields, as any sections surveyed will provide strong correlation between damage and larval presence. The presence of more mines than larvae has also been discussed in previous papers, suggesting that as larvae grow older, they may need to seek for younger tissues, and consequently, more than one mine can be created by the same individual [22]. However, as previously presented, older plants may yield lower infestation levels compared to those in early stages (i.e., flowering and early harvesting stages). Hence, in order to reduce damaging effects of *T. absoluta* during flowering and fruiting stages, the use of integrated pest management strategies is needed to reduce the damage of this invasive pest in Myanmar. Furthermore, it is highly imperative that plant protection and quarantine officers of Association of Southeast Asian Nations (ASEAN) member states coordinate their response to *T. absoluta* and build their capacity to monitor this pest and be prepared with suitable integrated pest management strategies when the pest arrives in their region(s).

## Figures and Tables

**Table 1:** Tomato sampling sites used during *Tuta absoluta* survey atthree locations and two cultivation methods in the South Shan State in Myanmar.

Cultivation	Location	Field	Villages	GPS Coordinates	Plant Stage	Variety/Type
Floating cultivation	Inle Lake Nyaungshwe	1	Nga Phe Chaung 0.14 ac	N 20° 31′ 1″ E 96° 53′ 59″	Late harvesting stage (5 months)	Princess (hybrid)/indeterminate
		2	Za Yiet Gyi 0.08 ac	N 20° 28′ 53″ E 96° 54′ 26″	Late harvesting stage (4 months)	Princess (hybrid)/indeterminate
		3	Kay Lar 0.08 ac	N 20° 30′ 10″ E 96° 55′ 0″	Late harvesting stage (4 months)	Princess (hybrid)/indeterminate
Land cultivation 1	Kalaw	1	- -	N 20° 35′ 17″ E 96° 36′ 31″	Last harvesting stage (5 months) *	unknown/indeterminate
		2	- 1.00 ac	N 20° 33′ 17″ E 96° 36′ 44″	Early harvesting stage (3.5 months)	909 (hybrid)/indeterminate
		3	Aung Ban^a^ 0.10 ac	N 20° 40′ 43″ E 96° 41′ 15″	Flowering stage (35–40 days)	cherry tomato (hybrid)/determinate
Land cultivation 2	Pin Ta Ya	1	Zaw Gyi 0.50 ac	N 20° 59′ 29″ E 96° 40′ 1″	Late harvesting stage (4.5 months)	909 (hybrid)/indeterminate
		2	Zaw Gyi 1.30 ac	N 20° 59′ 16″ E 96° 39′ 57″	Late harvesting stage (5 months)	909 (hybrid)/indeterminate
		3	Zaw Gyi 0.20 ac	N 20° 59′ 12″ E 96° 39′ 49″	Flowering stage (35 days)	909 (hybrid)/indeterminate

* Severely infected by late blight; ^a^ GAP farm; ^a^ ac = acreage.

**Table 2:** Mean *Tuta absoluta* infestation level and number of mine blotches per tomato leaflet at three locations under two different cultivation methods.

Cultivation	Location	Field	N	Mean Infestation (± SE) (%) (Min–Max Range)	Mean Number of Larvae (± SE) (Min–Max Range)	Mean Number of Mines/Leaf (± SE) (Min–Max Range)
Floating cultivation	Inle Lake Nyaungshwe	1	20	19.91 ± 3.85 (0–67)	0.00 ± 0.00(0–0)	4.75 ± 0.66(1–13)
		2	20	13.34 ± 2.64 (0–33)	0.35 ± 0.11(0–1)	2.00 ± 0.41(0–6)
		3	20	6.96 ± 2.42 (0–45)	1.35 ± 0.58(0–11)	4.60 ± 1.87(0–35)
Land cultivation 1	Kalaw	1	20	15.91 ± 2.71(0–36)	2.70 ± 1.13(0–16)	4.75 ± 1.63(0–27)
		2	20	81.59 ± 5.28(31–100)	14.35 ± 1.72(1–29)	34.00 ± 3.15(17–70)
		3	20	41.89 ± 5.49(7–91)	5.35 ± 0.93(0–14)	6.90 ± 1.00(1–17)
Land cultivation 2	Pin Ta Ya	1	20	5.73 ± 1.94(0–31)	0.45 ± 0.20(0–3)	2.25 ± 0.70(0–9)
		2	20	9.82 ± 3.03(0–61)	0.50 ± 0.15(0–2)	1.65 ± 0.42(0–7)
		3	20	5.11 ± 1.67(0–29)	0.75 ± 0.32(0–6)	2.35 ± 0.78(0–12)

## Data Availability

The data presented in this study are available on request from the corresponding author. The data can be accessed from https://worldveg.tind.io/.
